# Design of a Cylindrical Thermal Rotary Concentrator Based on Transformation Thermodynamics

**DOI:** 10.3390/ma18194440

**Published:** 2025-09-23

**Authors:** Ge Xia, Xing He, Yuhao Jia, Yan Zhu, Zegang Tian

**Affiliations:** College of Power Engineering, Naval University of Engineering, Wuhan 430033, China; xiage123123@163.com (G.X.); slone77777@163.com (Y.J.); 18835219305@139.com (Z.T.)

**Keywords:** transformation thermodynamics, thermal rotation, thermal concentration, temperature distribution

## Abstract

Thermal metamaterials are used in the design of thermal cloaks to achieve a series of functions, such as thermal invisibility and thermal concentration, representing a new approach for implementing energy transfer control in heat transfer processes. In this study, based on transformation thermodynamics, two functions, i.e., thermal rotation and thermal concentration, were set according to the time sequence and adopted the concept of spatial separation or space-sharing. Three cylindrical thermal rotary concentrators were designed by different spatial transformations. The numerical simulation results show that all three devices enable the concentration of heat flow in specific regions and alter the initial transfer direction of the heat flow. There is also no disturbance in the external heat flow transfer. These results effectively enrich the theory of transformation thermodynamics and provide different ideas for the subsequent study of thermal metamaterials.

## 1. Introduction

Thermal metamaterials have attracted considerable attention in recent years owing to their unique properties [1,2,3,4]. Based on the heat conduction equation, heat transfer is controlled by designing the thermophysical parameters of a medium to solve problems that conventional heat transfer control methods cannot address [5,6].

In 2008, Fan et al. [7], inspired by the concept of an electromagnetic invisibility cloak and referring to the theory of transformation optics, theoretically proposed a thermal invisibility cloak in the thermology field for the first time. The cloak protects the object inside from the heat flow from outside without causing any disturbance to the outside environment, as if the internal object did not exist. In the same year, Chen et al. [8] discussed a design method for thermal invisibility cloaks embedded inside anisotropic materials, demonstrating that coordinate transformation theory is still suitable. This study broadens the scope of the application of thermal invisibility cloaks and provides significant theoretical guidance for studying internal inhomogeneities. Li et al. [9] employed non-spherical nanoparticle composites with appropriate thermal and electrical conductivities to theoretically design a bifunctional cloak featuring electrical and thermal invisibility. Subsequently, Guenneau et al. [10] mathematically derived an expression for the required thermal conductivity based on the invariance of the thermal conductivity equation and proposed a method to construct a two-dimensional unstable thermal cloak and a thermal concentrator using a 20-layer concentric isotropic material. This result has attracted widespread research attention and has been reported by major media outlets such as the BBC. In this context, many theoretical designs for thermal metamaterial devices and related experimental verification schemes have been proposed [11,12,13,14,15]. The theory of transformation thermodynamics is applicable not only to the manufacturing of invisible devices but also to the design of various novel thermal metamaterials, such as thermal concentrators [16], thermal rotators [17], heat flow reversal devices [18], and thermal camouflage devices [19].

Topology optimization has recently been applied to the design of thermal cloaks. In 2018, Fujii et al. [20] proposed a topology optimization for thermal cloaks using a covariance matrix adaptation evolution strategy. This strategy has been applied to various thermal cloaking designs, including thermal–electrical biphysical cloaking [21,22,23]. Seitaro et al. [24] optimized the structural topology of the ITR-free thermal cloak by employing density-based topology optimization.

Most current studies on thermal metamaterials have focused on implementing a single function, such as thermal concentrators and thermal rotators. Inspired by the previous work on the design and research of functional devices [25], in this study, we designed a new thermal metamaterial device—a cylindrical thermal rotary concentrator, combining both thermal concentrator and thermal rotator functions in accordance with transformation thermodynamics. First, in addition to the function of thermal invisibility, the time sequence was set following the thermal rotation and thermal concentration functions. The idea of spatial separation was also adopted to design two cylindrical thermal rotary concentrators consisting of a three-layer medium, and the expression of the general solution for thermal conductivity in various regions of the two devices was derived. Subsequently, the space-sharing concept was adopted to combine thermal rotation and thermal concentration functions to simplify the structure of the device and space. We then proposed a spatial transformation method to accomplish the desired effect using only a two-layer medium and designed another cylindrical thermal rotary concentrator with a relatively simple structure. All three cylindrical thermal rotary concentrators were verified to be effective using finite element software COMSOL6.1. The three cylindrical thermal rotary concentrators designed in this study are named cylindrical thermal rotary concentrators No. 1, No. 2, and No. 3, following the sequence of their introduction for convenient differentiation.

## 2. The Design Method of Cylindrical Thermal Rotary Concentrator No. 1

**Figure 1:**
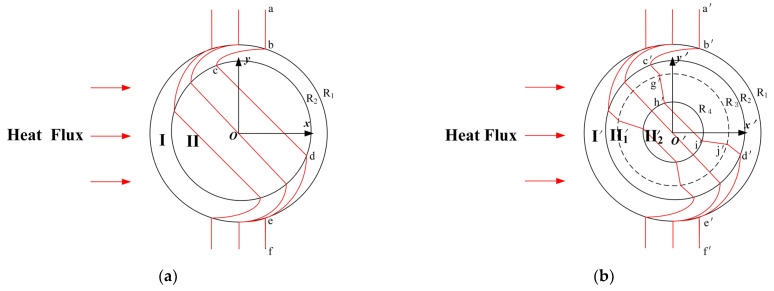
Schematic diagram of the spatial transformation process of the cylindrical thermal rotary concentrator No. 1. (**a**) Original space, (**b**) transformation space.

Figure 1a shows the schematic of a conventional cylindrical thermal rotator. Suppose that the heat flow is transferred horizontally from left to right, and when it enters the thermal rotator, the heat flow continuously rotates in Region I until it reaches the boundary $r=R_{2}$ and stops rotation. The rotation angle of the heat flow ranges from 0° at the initial state to the selected fixed angle (45° in this chapter). Meanwhile, the diagram shows that the isotherm becomes curved from the initial vertical curve a−b−c−d−e−f. The design method that includes a thermal concentrator in the central rotation area, Region II, is shown in Figure 1b, and the isotherm becomes $a^{\prime }-b^{\prime }-c^{\prime }-g^{\prime }-h^{\prime }-i^{\prime }-j^{\prime }-d^{\prime }-e^{\prime }-f^{\prime }$. In addition, the heat flow in the core area, Region ${{\mathrm{II}}^{\prime }}_{2}$, exhibits the effects of thermal rotation and thermal concentration with respect to the initial condition of the heat flow. The design principle of the entire spatial transformation is described as follows. Suppose that the conventional cylindrical thermal rotator shown in Figure 1a occupies the original space (x, y, z), and the thermal conductivity of the corresponding region is $\lambda_{1}$; the space where the cylindrical thermal rotary concentrator is located (Figure 1b) is the transformation space (x′, y′, z′), and the thermal conductivity of the corresponding region is $\lambda_{1}^{\prime }$. The transformation space can be divided into three different regions, according to the structural composition of the cylindrical thermal rotary concentrator: Region $\;I^{\prime }\left(R_{2}<r^{\prime }<R_{1}\right)$, Region ${{\mathrm{II}}^{\prime }}_{1}\left(R_{4}<r^{\prime }<R_{2}\right)$, and Region $\mathrm{II}\prime_{2}\left(0<r^{\prime }<R_{4}\right)$.

Given that the original space is a conventional cylindrical thermal rotator, we assumed that the initial thermal conductivity is $\lambda_{0}$. According to reference [26], the expressions of the thermal conductivity for each region in the original space can be obtained in the cylindrical coordinate system as follows:

Region I:

(1) $$\lambda_{1}=\left[\begin{array}{ccc} \left(1+2t_{1}\sin\theta \cos\theta +t_{1}^{2}\sin^{2}\theta \right) & -t_{1}^{2}\sin\theta \cos\theta -t_{1}\left(\cos^{2}\theta -\sin^{2}\theta \right) & 0\\ -t_{1}^{2}\sin\theta \cos\theta -t_{1}\left(\cos^{2}\theta -\sin^{2}\theta \right) & 1-2t_{1}\sin\theta \cos\theta +t_{1}^{2}\cos^{2}\theta & 0\\ 0 & 0 & 1 \end{array}\right]\lambda_{0}$$

where $t_{1}=\frac{\theta_{0}r}{R_{1}-R_{2}}=\frac{\pi }{4}\frac{r}{R_{1}-R_{2}}$.

Region II:

(2) $$\lambda_{1}=\lambda_{0}$$

In the transforming area, Region $I^{\prime }$, there is a one-to-one correspondence between the original space and the transformation space, and no transformation occurs, so the transformation equation between the original space and the transformation space is

(3) $$r^{\prime }=r,\theta^{\prime }=\theta ,z^{\prime }=z$$

Therefore, the thermal conductivity in the transformation region remains unchanged:

(4) $${\lambda^{\prime }}_{1}=\lambda_{1}==\left[\begin{array}{ccc} \left(1+2t_{1}\sin\theta \cos\theta +t_{1}^{2}\sin^{2}\theta \right) & -t_{1}^{2}\sin\theta \cos\theta -t_{1}\left(\cos^{2}\theta -\sin^{2}\theta \right) & 0\\ -t_{1}^{2}\sin\theta \cos\theta -t_{1}\left(\cos^{2}\theta -\sin^{2}\theta \right) & 1-2t_{1}\sin\theta \cos\theta +t_{1}^{2}\cos^{2}\theta & 0\\ 0 & 0 & 1 \end{array}\right]\lambda_{0}$$

For Region ${{\mathrm{II}}^{\prime }}_{1}$ formed by stretching the annular region $R_{3}<r<R_{2}$ in the original space, the equivalent stretch transformation equation is as follows:

(5) $$r^{\prime }=\frac{R_{2}-R_{4}}{R_{2}-R_{3}}r+\frac{R_{2}\left(R_{4}-R_{3}\right)}{R_{2}-R_{3}},\theta^{\prime }=\theta ,z^{\prime }=z$$

The thermal conductivity of the material in the Cartesian coordinate system is expressed as follows:

(6) $$\lambda_{1}^{\prime }=\left[\begin{array}{ccc} \frac{r^{\prime }-m_{2}}{r^{\prime }}\cos^{2}\theta^{\prime }+\frac{r^{\prime }}{r^{\prime }-m_{2}}\sin^{2}\theta^{\prime } & \left(\frac{r^{\prime }-m_{2}}{r^{\prime }}-\frac{r^{\prime }}{r^{\prime }-m_{2}}\right)\;sin\theta^{\prime }\cos\theta^{\prime } & 0\\ \left(\frac{r^{\prime }-m_{2}}{r^{\prime }}-\frac{r^{\prime }}{r^{\prime }-m_{2}}\right)\;sin\theta^{\prime }\cos\theta^{\prime } & \frac{r^{\prime }-m_{2}}{r^{\prime }}\sin^{2}\theta^{\prime }+\frac{r^{\prime }}{r^{\prime }-m_{2}}\cos^{2}\theta^{\prime } & 0\\ 0 & 0 & \frac{r^{\prime }-m_{2}}{m_{1}^{2}r^{\prime }} \end{array}\right]\lambda_{0}$$

where $m_{1}=\frac{R_{2}-R_{4}}{R_{2}-R_{3}}$, $m_{2}=\frac{R_{2}\left(R_{4}-R_{3}\right)}{R_{2}-R_{3}}$.

For Region ${{\mathrm{II}}^{\prime }}_{2}$ formed by compressing the circular region $0<r<R_{3}$ in the original space, the equivalent compression transformation equation is as follows:

(7) $$r^{\prime }=\frac{R_{4}}{R_{3}}r,\theta^{\prime }=\theta ,z^{\prime }=z$$

The thermal conductivity of Region ${{\mathrm{II}}^{\prime }}_{2}$ in the Cartesian coordinate system can be derived using the theory of transformation thermodynamics:

(8) $${\lambda^{\prime }}_{1}=\left[\begin{array}{ccc} 1 & 0 & 0\\ 0 & 1 & 0\\ 0 & 0 & (R_{3}/R_{4}{)}^{2} \end{array}\right]\lambda_{0}$$

Equations (4), (6), and (8) yield expressions of thermal conductivity in various regions of cylindrical thermal rotary concentrator No. 1, and the following numerical simulations are conducted to verify the above parameters. Consider that the entire computational domain is a 4 m × 4 m square area, and the other geometric dimensions of the device are $R_{1}=1.2\;m,\;R_{2}=1\;m,\;R_{3}=0.8\;m,\;R_{4}=0.4\;m$. The rotation angle is $\theta_{0}=\frac{\pi }{4}$. The top and bottom surfaces remain adiabatic, whereas the left and right surfaces retain a constant temperature, i.e., the left side at 400 K for the high-temperature side, and the right side at 300 K for the low-temperature side, so that the heat flow transfers from left to right. Copper was selected as the material in the area outside the thermal rotary concentrator. The thermal conductivities for Regions $I^{\prime }$, ${\mathrm{II}}_{1}^{\prime }$, and ${\mathrm{II}}_{2}^{\prime }$ are determined by Equations (4), (6), and (8), respectively.

**Figure 2:**
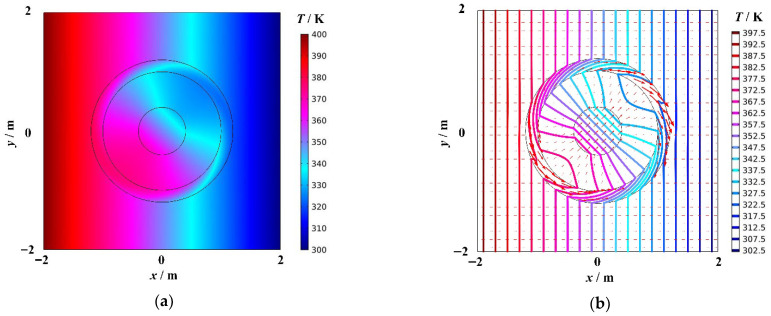
Cylindrical thermal rotary concentrator No. 1. (**a**) Temperature distribution diagram, (**b**) Diagram of the heat flow transfer and isotherms.

Figure 2a shows the temperature distribution of cylindrical thermal rotary concentrator No. 1, and Figure 2b shows the heat flow transfer and isotherm distribution. The red arrows represent the heat flow, and their size indicates the heat flow rate. The colored bold solid lines represent isotherms, and the temperature difference between two adjacent isotherms is 5 K. As shown in Figure 2b, there is no change in the temperature field outside the device. When the heat flow enters the device, the direction of the heat flow transfer in Region $I^{\prime }$ rotates continuously and stops when it reaches to the boundary $R_{2}$. At this point, the heat flow transfer direction is rotated 45°(anticlockwise) with respect to the initial transfer direction. After entering Regions ${{\mathrm{II}}^{\prime }}_{1}$ and ${{\mathrm{II}}^{\prime }}_{2}$, the temperature distribution and heat flow transfer in these regions undergo another change as a consequence of the energy concentration. The direction of the heat flow transfer in Region ${{\mathrm{II}}^{\prime }}_{1}$ is no longer 45° with respect to the initial transfer direction. In addition, according to the thermal concentrator design theory, the direction of the heat flow transfer in Region ${{\mathrm{II}}^{\prime }}_{2}$ is consistent with the direction of the heat flow transfer when the heat flow initially enters Region, i.e., it rotates anticlockwise by 45° with respect to the initial transfer direction. The density of isotherms in Region ${{\mathrm{II}}^{\prime }}_{2}$ is significantly greater than outside the device, and the arrows of the heat flow are noticeably larger compared to the arrows outside the device. The energy accumulation degree is related to $\frac{R_{3}}{R_{4}}$ by the principle of centralized design. In conclusion, cylindrical thermal rotary concentrator No. 1 encompasses three functions: invisibility, rotation, and concentration.

## 3. The Design Method of Cylindrical Thermal Rotary Concentrator No. 2

**Figure 3:**
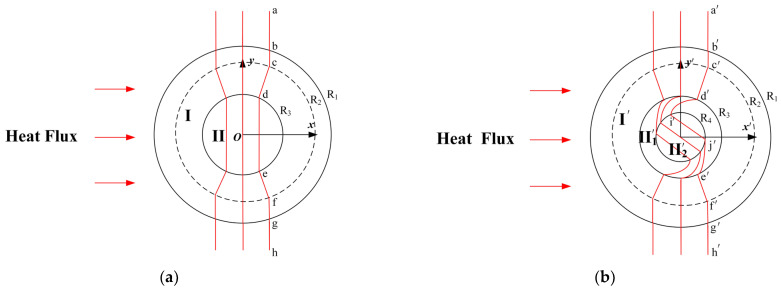
Schematic diagram of the spatial transformation process of the cylindrical thermal rotary concentrator No. 2. (**a**) Original space, (**b**) transformation space.

Similarly to the design method of cylindrical thermal rotary concentrator No. 1, it is possible to design another thermal rotary concentrator by switching the functional design order of rotation and concentration, i.e., cylindrical thermal rotary concentrator No. 2. Figure 3a shows a schematic diagram of a conventional cylindrical thermal concentrator. Suppose the heat flow is transferred from left to right; the heat flow is concentrated in Region II after entering the device. It is also evident from Figure 3 that the isotherm becomes curved from the initial vertical curve a−b−c−d−e−f−g−h. At this point, the design method that includes a thermal rotator in central energy concentration II is shown in Figure 3b, and the isotherm turns into $a^{\prime }-b^{\prime }-c^{\prime }-d^{\prime }-i^{\prime }-j^{\prime }-e^{\prime }-f^{\prime }-g^{\prime }-h^{\prime }$. In addition, the heat flow in Region ${{\mathrm{II}}^{\prime }}_{2}$ exhibits the effect of thermal rotation and thermal concentration with respect to the initial condition of the heat flow. The design principle of the entire spatial transformation is described as follows. Suppose that the conventional cylindrical thermal concentrator shown in Figure 3a occupies the original space (x, y, z), and the thermal conductivity of the corresponding region is $\lambda_{1}$; the space where the cylindrical thermal rotary concentrator is located, Figure 3b, is the transformation space (x′, y′, z′), and the thermal conductivity of the corresponding region is $\lambda_{1}^{\prime }$. The transformation space can be divided into three different regions according to the structural composition of the cylindrical thermal rotary concentrator: Region $\;I^{\prime }\left(R_{3}<r^{\prime }<R_{1}\right)$, Region ${{\mathrm{II}}^{\prime }}_{1}\left(R_{4}<r^{\prime }<R_{3}\right)$, and Region ${{\mathrm{II}}^{\prime }}_{2}\left(0<r^{\prime }<R_{4}\right)$.

Given that the original space is a conventional cylindrical thermal concentrator, suppose the initial thermal conductivity is $\lambda_{0}$. According to reference [27], the expressions of the thermal conductivity for each region in the original space can be obtained as follows:

Region І:

(9) $$\lambda_{1}=\left[\begin{array}{ccc} \frac{r^{\prime }-n_{2}}{r^{\prime }}\cos^{2}\theta^{\prime }+\frac{r^{\prime }}{r^{\prime }-n_{2}}\sin^{2}\theta^{\prime } & \left(\frac{r^{\prime }-n_{2}}{r^{\prime }}-\frac{r^{\prime }}{r^{\prime }-n_{2}}\right)\sin\theta^{\prime }\cos\theta^{\prime } & 0\\ \left(\frac{r^{\prime }-n_{2}}{r^{\prime }}-\frac{r^{\prime }}{r^{\prime }-n_{2}}\right)\sin\theta^{\prime }\cos\theta^{\prime } & \frac{r^{\prime }-n_{2}}{r^{\prime }}\sin^{2}\theta^{\prime }+\frac{r^{\prime }}{r^{\prime }-n_{2}}\cos^{2}\theta^{\prime } & 0\\ 0 & 0 & \frac{r^{\prime }-n_{2}}{n_{1}^{2}r^{\prime }} \end{array}\right]\lambda_{0}$$

where $n_{1}=\frac{R_{1}-R_{3}}{R_{1}-R_{2}}$; $n_{2}=\frac{R_{2}\left(R_{3}-R_{2}\right)}{R_{1}-R_{2}}$.

Region II:

(10) $$\lambda_{1}=\left[\begin{array}{ccc} 1 & 0 & 0\\ 0 & 1 & 0\\ 0 & 0 & (R_{2}/R_{3}{)}^{2} \end{array}\right]\lambda_{0}$$

In the transforming region, for Region $I^{\prime }$, there is a one-to-one correspondence between the original space and the transformation space, and no transformation occurs, so the transformation equation between the original space and the transformation space is

(11) $$r^{\prime }=r,\theta^{\prime }=\theta ,z^{\prime }=z$$

Therefore, the thermal conductivity in the transformation region remains unchanged:

(12) $${\lambda^{\prime }}_{1}=\lambda_{1}=\left[\begin{array}{ccc} \frac{r^{\prime }-n_{2}}{r^{\prime }}\cos^{2}\theta^{\prime }+\frac{r^{\prime }}{r^{\prime }-n_{2}}\sin^{2}\theta^{\prime } & \left(\frac{r^{\prime }-n_{2}}{r^{\prime }}-\frac{r^{\prime }}{r^{\prime }-n_{2}}\right)\sin\theta^{\prime }\cos\theta^{\prime } & 0\\ \left(\frac{r^{\prime }-n_{2}}{r^{\prime }}-\frac{r^{\prime }}{r^{\prime }-n_{2}}\right)\sin\theta^{\prime }\cos\theta^{\prime } & \frac{r^{\prime }-n_{2}}{r^{\prime }}\sin^{2}\theta^{\prime }+\frac{r^{\prime }}{r^{\prime }-n_{2}}\cos^{2}\theta^{\prime } & 0\\ 0 & 0 & \frac{r^{\prime }-n_{2}}{n_{1}^{2}r^{\prime }} \end{array}\right]\lambda_{0}$$

For Region ${{\mathrm{II}}^{\prime }}_{1}$ formed by rotating the annular region $R_{4}<r<R_{3}$ in the original space in a defined manner, the equivalent transformation equation is as follows:

(13) $$r^{\prime }=r,\theta^{\prime }=\theta +\theta_{0}\frac{f\left(R_{3}\right)-f\left(r\right)}{f\left(R_{3}\right)-f\left(R_{4}\right)},z^{\prime }=z$$

where $f\left(x\right)=x$; $\theta_{0}=\frac{\pi }{4}$.

The thermal conductivity of the material in the Cartesian coordinate system is expressed as follows:

(14) $$\;{\lambda^{\prime }}_{1}=\left[\begin{array}{ccc} 1+t_{2}^{2}\sin^{2}\theta^{\prime }+2t_{2}\sin\theta^{\prime }\cos\theta^{\prime } & -t_{2}^{2}\sin\theta^{\prime }\cos\theta^{\prime }-t_{2}\left(\cos^{2}\theta^{\prime }-\sin^{2}\theta^{\prime }\right) & 0\\ -t_{2}^{2}\sin\theta^{\prime }\cos\theta^{\prime }-t_{2}\left(\cos^{2}\theta^{\prime }-\sin^{2}\theta^{\prime }\right) & 1+t_{2}^{2}\cos^{2}\theta^{\prime }-2t_{2}\sin\theta^{\prime }\cos\theta^{\prime } & 0\\ 0 & 0 & (R_{2}/R_{3}{)}^{2} \end{array}\right]\lambda_{0}$$

where $t_{2}=\frac{\pi }{4}\frac{r^{\prime }}{R_{3}-R_{4}}$.

For Region ${{\mathrm{II}}^{\prime }}_{2}$ formed by rotating the annular region $0<r<R_{4}$ in the original space at a fixed angle (preset to 45°), the equivalent rotation transformation equation is

(15) $$r^{\prime }=r,\theta^{\prime }=\theta +\theta_{0},z^{\prime }=z$$

As a result, a computational shortcut yields a matrix:

(16) $${\lambda^{\prime }}_{1}=\lambda_{1}=\left[\begin{array}{ccc} 1 & 0 & 0\\ 0 & 1 & 0\\ 0 & 0 & (R_{2}/R_{3}{)}^{2} \end{array}\right]\lambda_{0}$$

**Figure 4:**
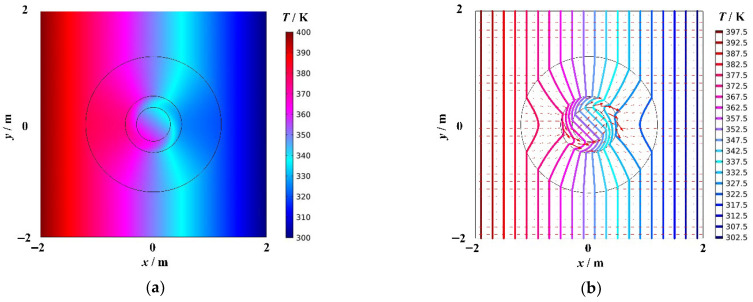
Cylindrical thermal rotary concentrator No. 2. (**a**) Temperature distribution diagram, (**b**) diagram of the heat flow transfer and isotherms.

Equations (12), (14), and (16) yield expressions of thermal conductivity in various regions of cylindrical thermal rotary concentrator No. 2. Numerical simulations were conducted using finite-element software COMSOL to verify the design parameters. Consider that the entire computational domain is a 4 m × 4 m square area, and the other geometric dimensions of the device are $R_{1}=1.2\;m,\;R_{2}=1\;m,\;R_{3}=0.5\;m,\;R_{4}=0.3\;m$. The rotation angle is $\theta_{0}=\frac{\pi }{4}$. The top and bottom surfaces remain adiabatic, whereas the left and right surfaces maintain a constant temperature, i.e., the left side at 400 K for the high-temperature side, and the right side at 300 K for the low-temperature side, so that the heat flow transfers from left to right. Copper was used as the material in the area outside the thermal rotary concentrator. The thermal conductivities of Regions $I^{\prime }$, ${\mathrm{II}}_{1}^{\prime }$, and ${\mathrm{II}}_{2}^{\prime }$ were determined using Equations (12), (14), and (16), respectively. The simulation results are shown in Figure 4:

Figure 4a shows the temperature distribution of cylindrical thermal rotary concentrator No. 2, and Figure 4b shows the heat flow transfer and isotherm distribution. The red arrows represent the heat flow, and their sizes indicate the heat flow rate. The colored bold solid lines represent isotherms, and the temperature difference between the two adjacent isotherms is 5 K. As shown in Figure 4b, there was no change in the temperature field outside the device. When the heat flow enters the device, it concentrates in Regions ${{\mathrm{II}}^{\prime }}_{1}$ and ${{\mathrm{II}}^{\prime }}_{2}$ in exchange for diluting the heat flow density in Region $I^{\prime }$. When the heat flow enters Region ${{\mathrm{II}}^{\prime }}_{1}$, its transfer direction continuously rotates and stops when it reaches the boundary $R_{4}$. At this point, the heat flow transfer direction was rotated 45° (anticlockwise) with respect to the initial transfer direction, i.e., the direction of the heat flow transfer in Region ${{\mathrm{II}}^{\prime }}_{2}$ rotates anticlockwise by 45° with respect to the initial transfer direction. The density of isotherms in Region ${{\mathrm{II}}^{\prime }}_{2}$ is significantly greater than that outside the device, and the heat flow arrows are noticeably larger than those outside the device. The degree of energy accumulation is related to $\frac{R_{2}}{R_{3}}$ by the principle of centralized design. In conclusion, cylindrical thermal rotary concentrator No. 2 exhibits three functions: invisibility, rotation, and concentration.

## 4. The Design Method of Cylindrical Thermal Rotary Concentrator No. 3

**Figure 5:**
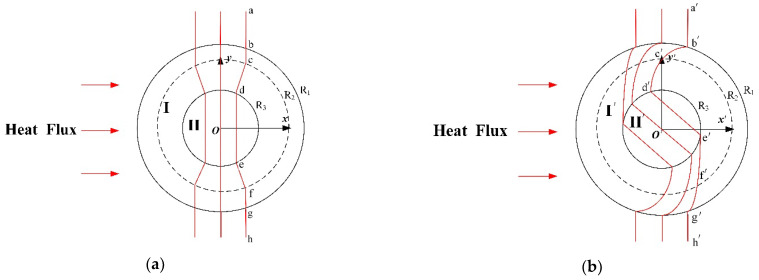
Schematic diagram of the spatial transformation process of cylindrical thermal rotary concentrator No. 3. (**a**) Original space, (**b**) transformation space.

Combining the functions of rotation and concentration, a spatial separation concept was adopted in the design of the previous two types of cylindrical thermal rotary concentrators, which required a three-layer medium. In contrast, the design of cylindrical thermal rotary concentrator No. 3 is based on the concept of space sharing by combining the functions of rotation and concentration, which can simplify the structure of the device and requires the design of only two media. Figure 5a is similar to Figure 3a, representing a schematic diagram of a conventional cylindrical thermal concentrator; its inner and outer diameters are $R_{3}$ and $R_{1}$, respectively. Similarly, the heat flow is concentrated in Region II after entering the device, and the isotherm also becomes curved from the initial vertical curve a−b−c−d−e−f−g−h. Consider the space in which the concentrator is located in the original space and includes a rotator of the same size as the concentrator, as shown in Figure 5b. At this point, the isotherm turns into $a^{\prime }-b^{\prime }-c^{\prime }-d^{\prime }-i^{\prime }-j^{\prime }-e^{\prime }-f^{\prime }-g^{\prime }-h^{\prime }$. Simultaneously, it is possible to concentrate the heat energy and rotate the heat flow at a fixed angle in Region ${\mathrm{II}}^{\prime }$ so that the device designed by this scheme is capable of both thermal rotation and thermal concentration. The design principle of the entire spatial transformation is described as follows. Suppose that the conventional cylindrical thermal concentrator shown in Figure 5a occupies the original space (x, y, z), and the thermal conductivity of the corresponding region is $\lambda_{1}$; the space where the cylindrical thermal rotary concentrator is located, Figure 5b, is the transformation space (x′, y′, z′), and the thermal conductivity of the corresponding region is $\lambda_{1}^{\prime }$. The transformation space can be divided into two different regions according to the structural composition of the cylindrical thermal rotary concentrator: Region $\;I^{\prime }\left(R_{3}<r^{\prime }<R_{1}\right)$ and Region ${\mathrm{II}}^{\prime }\left(0<r^{\prime }<R_{3}\right)$.

Given that the original space is a conventional cylindrical thermal concentrator, Equations (9) and (10) represent in detail the expressions for the thermal conductivity in various regions of the original space, which will not be repeated here. Similarly to Region $I^{\prime }$ formed by rotating the annular region $R_{3}<r<R_{1}$ in the original space in a defined manner, the equivalent transformation equation is

(17) $$r^{\prime }=r,\theta^{\prime }=\theta +\theta_{0}\frac{f\left(R_{1}\right)-f\left(r\right)}{f\left(R_{1}\right)-f\left(R_{3}\right)},z^{\prime }=z$$

where $f\left(x\right)=x$; $\theta_{0}=\frac{\pi }{4}$.

The material of the device is thermally conductive in all directions in the Cartesian coordinate system, which is represented as follows:

(18) $${\left({\lambda^{\prime }}_{1}\right)}_{xx}=\left[\frac{r^{\prime }-n_{2}}{r^{\prime }}\cos^{2}\theta^{\prime }+\left(\frac{r^{\prime }-n_{2}}{r^{\prime }}t_{3}^{2}+\frac{r^{\prime }}{r^{\prime }-n_{2}}\right)\sin^{2}\theta^{\prime }+2\frac{r^{\prime }-n_{2}}{r^{\prime }}t_{3}\sin\theta^{\prime }\cos\theta^{\prime }\right]\lambda_{0}$$

(19) $${\left({\lambda^{\prime }}_{1}\right)}_{xy}={\left({\lambda^{\prime }}_{1}\right)}_{yx}==\left\{\left[\frac{r^{\prime }-n_{2}}{r^{\prime }}\left(1-t_{3}^{2}\right)-\frac{r^{\prime }}{r^{\prime }-n_{2}}\right]\sin\theta^{\prime }\cos\theta^{\prime }-\frac{r^{\prime }-n_{2}}{r^{\prime }}t_{3}\left(\cos^{2}\theta^{\prime }-\sin^{2}\theta^{\prime }\right)\right\}\lambda_{0}$$

(20) $${\left({\lambda^{\prime }}_{1}\right)}_{yy}=\left[\frac{r^{\prime }-n_{2}}{r^{\prime }}\sin^{2}\theta^{\prime }+\left(\frac{r^{\prime }-n_{2}}{r^{\prime }}t_{3}^{2}+\frac{r^{\prime }}{r^{\prime }-n_{2}}\right)\cos^{2}\theta^{\prime }-2\frac{r^{\prime }-n_{2}}{r^{\prime }}t_{3}\sin\theta^{\prime }\cos\theta^{\prime }\right]\lambda_{0}$$

(21) $${\left({\lambda^{\prime }}_{1}\right)}_{zz}=\frac{r^{\prime }-n_{2}}{n_{1}^{2}r^{\prime }}\lambda_{0}$$

(22) $${\left({\lambda^{\prime }}_{1}\right)}_{xz}={\left({\lambda^{\prime }}_{1}\right)}_{zx}={\left({\lambda^{\prime }}_{1}\right)}_{yz}={\left({\lambda^{\prime }}_{1}\right)}_{zy}=0$$

where $t_{3}=\frac{\pi }{4}\frac{r^{\prime }}{R_{1}-R_{3}}$.

For Region ${{\mathrm{II}}^{\prime }}_{2}$ formed by rotating the annular region $0<r<R_{3}$ in the original space at a fixed angle (preset to 45°), the equivalent rotation transformation equation is

(23) $$r^{\prime }=r,\theta^{\prime }=\theta +\theta_{0},z^{\prime }=z$$

As a result, a computational shortcut yields the following matrix:

(24) $${\lambda^{\prime }}_{1}=\lambda_{1}=\left[\begin{array}{ccc} 1 & 0 & 0\\ 0 & 1 & 0\\ 0 & 0 & {\left(R_{2}/R_{3}\right)}^{2} \end{array}\right]\lambda_{0}$$

Equations (18)–(22) and (24) yield expressions for the thermal conductivity in various regions of cylindrical thermal rotary concentrator No. 3. Numerical simulations were conducted using finite-element software COMSOL to verify the design parameters. Similarly to the conditions of the first two simulations, the entire computational domain is a 4 m × 4 m square area, and the other geometric dimensions of the device are $R_{1}=1.2\;m,\;R_{2}=1\;m,\;R_{3}=0.5\;m$. The rotation angle is $\theta_{0}=\frac{\pi }{4}$. The top and bottom surfaces stay adiabatic, whereas the left and right surfaces keep a constant temperature, i.e., the left side remains at 400 K as the high-temperature side, and the right side remains at 300 K as the low-temperature side, such that the heat flow is transferred from left to right. Copper was selected as the material in the area outside the thermal rotary concentrator.

**Figure 6:**
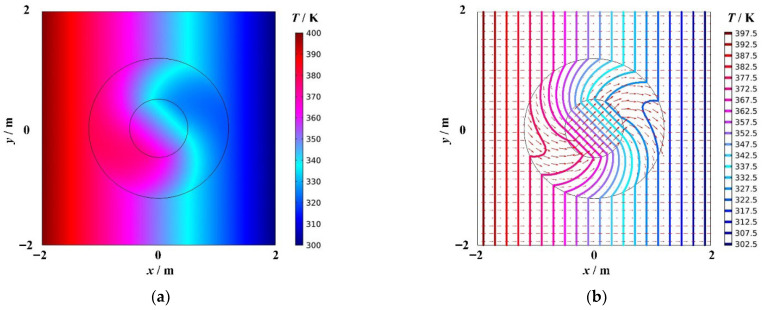
Cylindrical thermal rotary concentrator No. 3. (**a**) Temperature distribution diagram, (**b**) diagram of heat flow transfer and isotherms.

The thermal conductivities of Regions $I^{\prime }$ and ${\mathrm{II}}^{\prime }$ are determined by Equations (18)–(22) and (24), respectively. The simulation results are shown in Figure 6.

Figure 6a shows the temperature distribution of cylindrical thermal rotary concentrator No. 3, and Figure 6b shows the heat flow transfer and isotherm distribution. The red arrows represent the heat flow, and their size indicates the heat flow rate. The colored bold solid lines represent isotherms, and the temperature difference between the two adjacent isotherms is 5 K. As shown in Figure 6b, there is no change in the temperature field outside the device. When the heat flow enters Region $I^{\prime }$, dual functions of rotation and concentration induce a special change in the transfer direction and rate of the heat flow. When the heat flow entered Region ${\mathrm{II}}^{\prime }$, the heat flow transfer direction rotated anticlockwise by 45° with respect to the initial transfer direction. In addition, the density of isotherms in Region ${\mathrm{II}}^{\prime }$ is significantly greater than that outside the device, and the heat flow arrows are noticeably larger than the arrows outside the device. The energy accumulation degree is related to $\frac{R_{2}}{R_{3}}$ by the principle of centralized design. In conclusion, cylindrical thermal rotary concentrator No. 3 exhibits three functions: invisibility, rotation, and concentration.

## 5. Summary

In this study, based on transformation thermodynamics, we designed three different thermal rotary concentrators by combining two functions, i.e., thermal concentration and thermal rotation, and using different spatial transformation methods. The designed devices were verified using finite-element software COMSOL, and all devices simultaneously exhibited three functions: invisibility, rotation, and concentration.

From the models and simulations, we learn that the performance (e.g., temperature gradient in the core) is predominantly governed by the concentration transformation, while the rotation accuracy is determined by the initial rotation angle design, confirming the independent controllability of these parameters. More importantly, we found that the choice of transformation sequence (Cylindrical Thermal Rotary Concentrator NO. 1: rotate-then-concentrate vs. Cylindrical Thermal Rotary Concentrator NO. 2: concentrate-then-rotate) can influence the complexity and anisotropy of the required material parameters, which has implications for practical fabrication. Cylindrical Thermal Rotary Concentrator NO. 3, employing a concurrent transformation strategy, presents a more elegant and potentially more compact solution, achieving both functions within a shared spatial domain, which might simplify future experimental realization.

This design extends the application of transformation thermodynamics and provides different ideas for further design of thermal metamaterials. The wider applicability of these devices is promising in advanced thermal management systems. For instance, in electronic cooling, they could be used to redirect and focus heat from a CPU hotspot towards a dedicated heat sink, mitigating thermal throttling. In energy harvesting, they could enhance the efficiency of thermoelectric generators by concentrating waste heat onto a smaller generator area while orienting the heat flow optimally.

## Data Availability

The original contributions presented in this study are included in the article. Further inquiries can be directed to the corresponding authors.
